# Supplementary material for: Role and mechanism of NCAPD3 in promoting malignant behaviors in gastric cancer
Source: Front Pharmacol. 2024 Apr 22;15:1341039. doi: 10.3389/fphar.2024.1341039 (PMC11070777; doi:10.3389/fphar.2024.1341039)
Supplement: Supplementary file 11 [file DataSheet2.ZIP › GSEA/Transcipt factor/my_analysis.Gsea.1599461814734/FREAC2_01.html]

Details for gene set FREAC2\_01[GSEA]

|  || Dataset | filtered\_dataset.sample\_info.cls#WT\_versus\_NCAPD3\_MUT |
| Phenotype | sample\_info.cls#WT\_versus\_NCAPD3\_MUT |
| Upregulated in class | NCAPD3\_MUT |
| GeneSet | FREAC2\_01 |
| Enrichment Score (ES) | -0.41901565 |
| Normalized Enrichment Score (NES) | -2.1115966 |
| Nominal p-value | 0.0051150895 |
| FDR q-value | 0.040675294 |
| FWER p-Value | 0.319 |
Table: GSEA Results Summary

  

Fig 1: Enrichment plot: FREAC2\_01      
 Profile of the Running ES Score & Positions of GeneSet Members on the Rank Ordered List

  

| SYMBOL | TITLE | RANK IN GENE LIST | RANK METRIC SCORE | RUNNING ES | CORE ENRICHMENT || 1 | 51585 | PCF11 | 3 | 1.212 | 0.0634 | No |
| 2 | 51134 | CEP83 | 19 | 1.038 | 0.1088 | No |
| 3 | 23338 | JADE2 | 71 | 0.855 | 0.1182 | No |
| 4 | 4281 | MID1 | 294 | 0.595 | -0.0102 | No |
| 5 | 79047 | KCTD15 | 469 | 0.493 | -0.1093 | No |
| 6 | 3479 | IGF1 | 511 | 0.469 | -0.1135 | No |
| 7 | 4784 | NFIX | 567 | 0.439 | -0.1296 | No |
| 8 | 26100 | WIPI2 | 601 | 0.423 | -0.1305 | No |
| 9 | 149076 | ZNF362 | 680 | 0.388 | -0.1660 | No |
| 10 | 8904 | CPNE1 | 814 | 0.308 | -0.2455 | No |
| 11 | 7067 | THRA | 976 | -0.392 | -0.3407 | No |
| 12 | 23764 | MAFF | 1039 | -0.436 | -0.3619 | No |
| 13 | 2012 | EMP1 | 1119 | -0.494 | -0.3923 | Yes |
| 14 | 64795 | RMND5A | 1142 | -0.508 | -0.3807 | Yes |
| 15 | 7074 | TIAM1 | 1143 | -0.508 | -0.3532 | Yes |
| 16 | 639 | PRDM1 | 1159 | -0.523 | -0.3357 | Yes |
| 17 | 2119 | ETV5 | 1176 | -0.545 | -0.3178 | Yes |
| 18 | 6310 | ATXN1 | 1177 | -0.547 | -0.2881 | Yes |
| 19 | 153222 | CREBRF | 1187 | -0.559 | -0.2644 | Yes |
| 20 | 10150 | MBNL2 | 1217 | -0.591 | -0.2534 | Yes |
| 21 | 2355 | FOSL2 | 1262 | -0.648 | -0.2501 | Yes |
| 22 | 54800 | KLHL24 | 1287 | -0.677 | -0.2308 | Yes |
| 23 | 3084 | NRG1 | 1320 | -0.739 | -0.2140 | Yes |
| 24 | 54790 | TET2 | 1364 | -0.842 | -0.1995 | Yes |
| 25 | 10397 | NDRG1 | 1380 | -0.925 | -0.1603 | Yes |
| 26 | 4919 | ROR1 | 1385 | -0.954 | -0.1116 | Yes |
| 27 | 26959 | HBP1 | 1395 | -1.073 | -0.0600 | Yes |
| 28 | 51274 | KLF3 | 1406 | -1.296 | 0.0029 | Yes |
Table: GSEA details [plain text format]

  

Fig 2: FREAC2\_01      
 Blue-Pink O' Gram in the Space of the Analyzed GeneSet

  

Fig 3: FREAC2\_01: Random ES distribution      
 Gene set null distribution of ES for **FREAC2\_01**
